# Effect of biofabricated zinc oxide nanoparticles on callus and in vitro regenerated shoots of *Reseda lutea*, and assessment of biochemical responses, polyphenolic content, and genetic stability

**DOI:** 10.1080/15592324.2025.2558871

**Published:** 2025-09-17

**Authors:** Salim Khan, Abdulrahman Al-hashimi, Mohammad Nadeem, Mohamed Tarroum, Abdalrhaman M. Salih, Norah Abdullah Alsharif, Fahad Al-Qurainy

**Affiliations:** Department of Botany and Microbiology, College of Science, King Saud University, Riyadh, Saudi Arabia

**Keywords:** Zinc nanoparticles, polyphenol, antioxidant response, genome size, callus

## Abstract

The conservation of rare and endangered plant species has progressed with the advent of nanotechnology, enabling their large-scale production with desirable traits. The present study was focused on the synthesis of zinc oxide nanoparticles (ZnO-NPs) using the aqueous extract of *Convolvulus arvensis* and their characterization using various techniques (UV spectra, FTIR, transmission electron microscopy (TEM), and zeta potential), and further, their impact was assessed on callus and *in vitro* raised shoots of *Reseda lutea*. Low concentrations of ZnO-NPs (15 and 30 mg/L) increased the fresh weight of shoots by 35.38% and 17.43%, respectively. In contrast, a high concentration of ZnO-NPs (60 mg/L) in MS medium resulted in a 29% decrease in shoot biomass. The different concentrations of ZnO-NPs (15, 30, and 60 mg/L) increased the callus biomass by 70.7%, 62.6%, and 24.8%, respectively, compared to the control. The total phenolic content (TPC) and flavonoid content (TFC) in both regenerated stages were varied, and they were increased in callus by 15.5% with 60 mg/L of ZnO-NPs, whereas TPC and TFC were reduced in shoot, and a greater reduction was observed in TFC with the same concentration of ZnO-NP treatment than the control. The biochemical analysis performed on callus and shoot revealed a dose-dependent accumulation of proline and TBARS content. The accumulation of total soluble protein improved in both regeneration stages, and its content varied with different treatment doses of ZnO-NPs. A close relationship was observed in protein accumulation by 26.24%, and chlorophyll contents by 36.4% in shoots with 15 mg/L ZnO-NPs than the control, while both parameters decreased with 60 mg/L ZnO-NPs. The activities of antioxidant enzymes, including GR, SOD, and APX, varied under different treatment doses of ZnO-NPs. The flow cytometry (FCM) results of callus and shoot with ZnO-NPs treatment confirmed the genetic stability by genome size (2C DNA content). The results of this study show that biogenic ZnO-NPs positively influence various attributes of the callus and shoot stages and may support the mass production of *R. lutea* with abiotic stress tolerance.

## Graphical abstract

**Figure:**
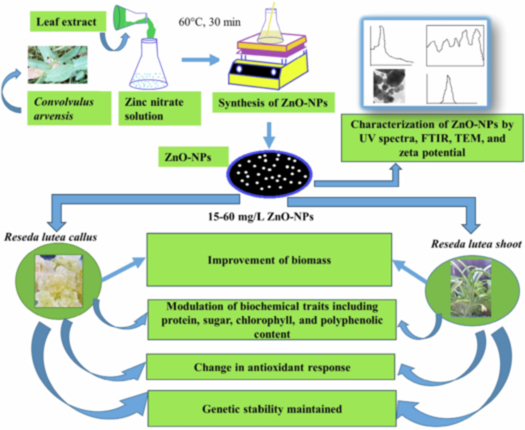

## Introduction

*Reseda lutea* L. is a medicinal plant of the family Resedaceae. The Resedaceae is a comparatively small family with only a few genera, including *Randonia*, *Reseda*, *Ochradenus*, *Oligomeris*, *Caylusea*, and *Sesamoides.*^1^ Few species of the genus *Reseda* have been documented, viz. *R. lutea*, *R. alba*, *R. aucheri*, *R. muricata*, *R. arabica*, *R. sphenocleoides*, and *R. pentagyna* in Saudi Arabia. *R. lutea* is a biennial or perennial herbaceous plant that thrives in various places, such as uncultivated fields, roadsides, and rocky slopes. Based on the literature review, this plant species is considered rare and restricted to some regions of Saudi Arabia.^2^ However, it is found in many temperate zones worldwide.^3^ This plant species has been used for many purposes, including apiculture, as a food source for cattle, and to prevent soil erosion due to its fast-growing roots. The roots of *R. lutea* contain diuretic and antidiarrheal characteristics, antibacterial, anti-inflammatory, and tumor suppression activities.^4-6^ The aerial parts of *R. lutea* have important flavonol glycosides (kaempferol).^7^ As in our previous research on *R. lutea*, the molecular phylogenetic study was conducted using the internal transcribed spacer sequence of rDNA (nr-DNA-ITS) and found its closeness to other species of the family Resedaceae.^8^ Additionally, the genome size of *R. lutea* was determined through flow cytometry and compared against results based on k-mer analysis.

Recent developments in nanotechnology have had a substantial impact on the fields of biomedical and plant sciences through the synthesis of surface-functionalized materials intended to interact with biological systems. For instance, through precise molecular interactions, surface-engineered biomaterials have demonstrated promise in controlling cellular responses and enhancing hemostatic capabilities.^9^ These biomimetic concepts can be applied to plant systems, where nanoparticles—particularly those with certain physicochemical characteristics—interact with cells to control metabolite accumulation, growth, and differentiation. Furthermore, research on the pharmacokinetics of compounds derived from plants, such as senkyunolide I, has highlighted the significance of transport dynamics and the biochemical environment, underscoring the intricate relationship between metabolism and bioavailability in plant-based treatments.^10^ These perspectives emphasize that to fully utilize the biochemical potential of therapeutic plants, in vitro cultivation conditions are ideal and necessary. The importance of phytochemical diversity and its relationship to medicinal efficacy are further supported by a recent critical evaluation of *Ruta graveolens*, which calls for innovative biotechnological strategies to improve the synthesis of bioactive compounds.^11^ Building on these ideas, the current study investigated how biogenic zinc oxide nanoparticles can be utilized on *R. lutea* to increase secondary metabolite production and propagation efficiency. The absence of a control treatment with equal amounts of ionic zinc (such as Zn⁺² from a salt solution) to distinguish the effects of nanoparticle-specific treatment from those of general micronutrient supplementation is an issue in the experimental design. Although the growth and metabolic alterations are attributed to ZnO-NPs exposure, it is unclear whether the nanoparticulate form or just the additional Zn nutrient is responsible for these benefits at low doses. Zinc is a necessary element in plant systems; thus, addressing a zinc deficit rather than a nanospecific signaling function may lead to better growth. Earlier research has demonstrated that ZnO nanoparticles and bulk ZnO and Zn⁺² ions have different phytotoxicities.^12^

The multiplication of rare plant species using nanobiotechnology is an emerging field that integrates nanotechnology and biotechnology to enhance plant propagation, introduce abiotic tolerance, and promote conservation. Recent developments in nanotechnology have facilitated the synthesis of nanoparticles, which can be broadly used for various purposes.^13^ Some nanoparticles act as nanopesticides to control plant diseases caused by various pathogens.^14-16^ Many nanoparticles have been used as nanofertilizers to enhance nutrient uptake and increase crop yield parameters.^17^^,^^18^ Nanoparticles are assemblies of atoms or molecules, generally small in size with large surface areas, that significantly change their physicochemical properties compared to bulk materials**.**^19^ The synthesis of nanoparticles using plant extracts is an eco-friendly and cost-effective approach using green nanotechnology. *In vitro* methods, including the application of nanoparticles in Murashige and Skoog media (MS media) for the enhancement of growth,^20^ are increasing due to their advantages over traditional propagation techniques. These nanoparticles have shown important potential in augmenting the efficiency of plant tissue culture methods because of their unique properties, such as reduced toxicity, biocompatibility, and ability to act together at the cellular level.

Zinc oxide nanoparticles (ZnO-NPs) play an important role in tissue culture for increasing plant growth, controlling contamination, and stress tolerance.^21-23^ Their incorporation into *in vitro* protocols can reform plant biotechnology, making micropropagation more competent and sustainable. Zinc (Zn) plays an important role in DNA synthesis and metabolism, indicating that its absence can have great cellular consequences.^24^ All living things require zinc, a micronutrient that plays a crucial role in defense, growth, and development. The chlorosis, stunted development, and leaf abnormalities are common signs of zinc deficiency.^25^ Many enzymes play an important role in metabolism, which contain zinc as a cofactor.^26^ The standardization of mineral nutrition in plants is an important strategy to improve their health and yield traits. Zinc participates in plant defense mechanisms against various pathogens and herbivores.^27^

In the previous study, plant regeneration in *R. lutea* was performed from adventitious buds (direct organogenesis),^28^ and there have been no studies performed on plant multiplication using indirect organogenesis along with nanoparticles. However, compared to the indirect method, direct organogenesis generally yields fewer roots or shoots per explant, and the overall rate of multiplication is frequently lower. This can be enhanced by the introduction of biogenic nanoparticles, mainly zinc oxide nanoparticles, at different stages of regeneration using tissue culture. Many studies have shown that zinc oxide nanoparticles have a promoting effect when applied at low doses; however, excessive doses can lead to growth retardation and induce stress in plants.^29-32^ ZnO-NPs can produce reactive oxygen species (ROS), such as hydroxyl radicals (OH^−^), hydrogen peroxide (H_2_O_2_), and superoxide radicals (O^2^^−^),^33^ which can have both positive and negative effects.^34^ The application of different concentrations of nanoparticles produces various effects on plant calluses and organs.^35^^,^^36^

A plant callus is a group of undifferentiated and organized structures formed when different kinds of plant explants are cultured *in vitro* under defined conditions with plant growth regulators. The callus can be induced from various explants of plants, such as root, shoot, and leaf.^37-39^ The callus development is a key step in plant tissue culture, which acts as an intermediate stage to produce the shoots and further whole plant regeneration. Different types of explants, culture conditions, regeneration media components, genotypes, and physiological states of the mother plant affect the frequency of regeneration.^40^ The regenerated shoots from the callus can vary due to differences in their developmental stage, function, and structure. Considering the potential use of zinc as a micronutrient in the literature, we present the environmentally friendly phytomediated synthesis of ZnO-NPs using aqueous extracts of *Convolvulus arvensis* (herbaceous weed) as a reducing agent and their application on *in vitro* raised calluses and shoots of *R. lutea* to find the optimum dose for mass production.

## Materials and methods

### Plant collection

The mature seeds of *R. lutea* and leaves of *C. arvensis* were collected from Abha and Al-Heir in Saudi Arabia. The plant was identified by a botanist at the Botany and Microbiology Department, King Saud University. The collected seeds of *R. lutea* were stored at 4 °C to maintain their viability and for long-term preservation. The identification of the plant was also confirmed by a molecular marker, the internal transcribed spacer sequence of rDNA.

### DNA extraction and molecular identification

The genomic DNA of high quality was extracted from the leaves of *R. lutea* and *C. arvensis* using Khan et al. protocol.^41^ The purified DNA was diluted in double-distilled water to set up the PCR reaction with specific primers (ITS1 reverse 5′-TCCTCCGCTTATTGATATGC-3′ and ITS4 forward 5′-GTCCACTGAACCTTATCATTTAG-3′ designed from Macrogen, South Korea). The following parameters were used to conduct the PCR reaction in a Techne thermal cycler: the first denaturation at 94 °C for 5 min was performed. The segment denaturation was carried out at 94 °C for 1 min, 50 °C for 30 s for annealing, and 72 °C for 1 min for extension for 35 cycles. The final extension at 72 °C for 5 min was carried out in the last cycle. The PCR results were sequenced at Macrogen in Seoul, South Korea.

### Seed sterilization and germination

The mature seeds were rinsed five times with distilled water after being treated with 50% Clorox for 10 min. The seeds were soaked for 24 h at room temperature in autoclaved double-distilled water to promote germination. Following the sterilization, the seeds were placed on Murashige and Skoog (MS) medium, which was acquired from Sigma and enhanced with various ingredients, such as 0.8% (w/v) plant agar and 3.0% (w/v) sucrose. The seed germination was accomplished under optimal conditions at a temperature of 25 ± 2 °C in dark conditions.

## Induction of callus on MS media

The MS medium (Murashige & Skoog, 1962) supplemented with vitamins in powder form (Sigma-Aldrich Chemicals Company) was used for the induction of callus with plant growth regulators (PGRs). After 5 weeks of germination, cotyledons were collected from seedlings and subcultured on MS media to raise the callus under dark conditions at the optimum temperature (22 °C ± 2 °C). The phytohormone combination (0.1, 0.2, and 0.3 mg/L 2,4-dichlorophenoxyacetic acid with BAP-0.1 mg/L) was used for the induction of callus. Sucrose was employed at a concentration of 30 g/L as the carbon source, and 0.8% agar was added to solidify the MS medium. The application of MS medium provided the indispensable nutrients required for shoot differentiation and development. The MS medium was autoclaved for 30 min at 121 °C and 1.05 kg/cm^2^ (15−20 psi).

### Shoot induction

The callus produced on MS media was cultured for shoot induction (organogenesis) with plant growth regulator (PGR), including 0.1, 0.2, 0.3, and 0.4 mg/L BAP. Once shoots were induced with BAP on MS media, they were transferred to MS media for shoot multiplication. The cultures were grown under optimum conditions (22 ± 2 °C) for four weeks for multiple shoot production.

### Shoot multiplication

The MS medium was supplemented with the optimal concentration of cytokinin, as BAP was previously determined to be effective for shoot induction. The cultures were grown at 22 ± 2 °C under a 16-h photoperiod with light intensity of nearly 40–60 µmol m⁻² s⁻¹ for 4 weeks.

### Preparation of plant extract

Young leaves of *C. arvensis* were used for the fabrication of nanoparticles. *C. arvensis* was identified using the internal transcribed spacer sequence of nuclear ribosomal DNA (nrDNA-ITS). The fresh leaves (10 g) of *C. arvensis* were made into powder with liquid nitrogen, mixed with double-distilled water (150 ml), and kept in a water bath at 100 °C for 30 min. The mixture was cooled and filtered through Whatman filter paper. Freshly prepared aqueous extract of *C. arvensis* was employed for the synthesis of zinc oxide nanoparticles.

### Biofabrication of ZnO-NPs and characterization

ZnO-NP fabrication was conducted according to the protocol followed by Al-Qurainy et al.^21^ A total of 200 mL of leaf extract of *C. arvensis* was mixed with 0.05 M Zn (NO_3_)_2_ solution at 60 °C, with constant stirring for 10 h. The synthesis of nanoparticles in the mixture was validated by UV–spectra (NanoDrop spectrophotometer). The nanoparticles were taken in pellet form upon centrifugation at 8000  rpm at 4 °C for 10 min. The pellet was washed with 70% alcohol and dried at 40 °C for 12 h. The synthesized ZnO-NPs were calcined at 500 °C for 5 h.

The calcined ZnO-NPs (5 mg) were dissolved in 5mL of double-distilled water for analysis. To check the optical properties, the absorption spectra of the ZnO-NPs were measured using a UV–vis spectrophotometer (Nanodrop spectrophotometer 8000) within a wavelength range of 200–800 nm. The surface properties of the functional groups and biomolecules attached to the ZnO-NPs were analyzed by FT-IR spectroscopy (Shimadzu) at a frequency of 4000−400 cm⁻¹. The particle stability was determined by zeta potential (Malvern Zetasizer, Malvern Instruments Ltd., Malvern, UK). Furthermore, the evaluation of the size of ZnO-NPs was performed using transmission electron microscopy (TEM) (JEM−1400).

### Callus and shoot treatment with biogenic ZnO-NPs

The callus and shoot (2-month-old) raised on MS media were used for the ZnO-NPs treatment. Three concentrations of ZnO-NPs, including 15, 30, and 60 mg/L, were added into MS medium in which callus as well as shoot were grown for 4 weeks under a photoperiod of 16 h light and 8 h darkness. The callus and shoot biomass were recorded after 4 weeks of culture on MS medium. Similarly, the shoot length, number of shoots, and shoot biomass were recorded under the above treatments and compared with the control as well as among the treatments.

### Study on biochemical parameters

#### Total chlorophyll content estimation in shoots

The content of total chlorophyll was estimated using the method as developed by Lichtenthaler.^42^ The sample (0.1 g) was chopped into small pieces and transferred into a 2 ml Eppendorf tube. Next, 100% acetone (2 ml) was employed for the extraction of total chlorophyll. The absorbance was measured at 663 nm and 645 nm using UV–vis spectrophotometer:

Chlorophyll a = 12.25 A663.2 − 2.79 A646.8,

Chlorophyll b = 21.50 A646.8 − 5.10 A663.2,

The chlorophyll content (a + b) was expressed in µg/g FW.

#### Estimation of total crude protein content

The total protein content was determined in callus and shoots by Nanodrop spectrophotometer at 280 nm, as followed by Khan et al.^43^ The fresh samples of callus and shoot (0.4 g) were ground into powder with liquid nitrogen. The samples were taken into an Eppendorf tube, and 2 mL of phosphate buffer (pH = 7.2) was added to it and mixed well. The mixture was centrifuged at 7000 rpm to collect the supernatant, and the absorbance was taken to calculate the protein concentration.

#### Proline content

For the measurement of proline, the fresh samples of shoot and callus were obtained following the method of Hanson et al.^44^ The sample of 0.3 g was ground in 5 mL of 3% aqueous sulfosalicylic acid. The mixture was centrifuged, and the supernatant was taken for use in the consequent reaction setup. 2 mL of supernatant was taken in a glass test tube in which glacial acetic acid (2 mL) and acidic ninhydrin reagent (2 mL) were added, and then kept on a water bath at 100 °C for 30 min. The mixture was cooled by keeping it on ice. The toluene (4 mL) was added to the above reaction mixture, and the color intensity of the sample was taken at 520 nm. The content of proline was determined by quantifying the absorbance of chromatophores present in toluene at 520 nm, and proline was expressed in µg/g fresh weight.

#### Estimation of TBAR content

TBARS content was assessed in fresh callus and shoots of *R. lutea* using the protocol of Cakmak and Horst.^45^ The samples (0.25 g) were made into powder in liquid nitrogen. The powder was taken into a 15 mL Falcon tube, to which 10 mL trichloroacetic acid (0.1%, w/v) was added. The centrifugation was performed at 12,000 rpm to collect the supernatant. The supernatant was transferred into a glass tube to initiate the reaction by mixing 2 mL of the sample with 4 mL of 0.5% (w/v) thiobarbituric acid (TBA) in 20% (w/v) trichloroacetic acid (TCA). The mixture was then incubated in a water bath at 90 °C for 30 min. TBARS content was measured from the absorbance taken at 532 and 600 nm using a spectrophotometer.

$$\mathrm{TBARS}\;(\mathrm{nmolg}-1\mathrm{fw})=\frac{(A532-A600)\;\times \;V\;\times \;100}{155\times \mathrm{extinction}\;\mathrm{coefficient}\;\times \;w\;\times \;1},$$

A532 represents the absorbance at 532 nm, and A600 represents the absorbance at 600 nm, V = extraction volume, and W = fresh weight of tissue.

#### Estimation of antioxidant enzyme activity

##### Superoxide dismutase (EC 1.15.1.1)

The activity of superoxide dismutase (SOD) was estimated using the protocol of Dhindsa et al.^46^ The enzyme was isolated from the fresh sample (0.25 g) with the extraction buffer (phosphate buffer, pH 7.4). The color reduction (50%) was used to determine the SOD activity and presented as EU mg^−1^ protein min^−1^.

##### Glutathione reductase (EC 1.6.4.2)

The Rao^47^ protocol was employed for the assessment of the activity of glutathione reductase (GR) at different treatments of ZnONPs. The fresh sample (0.3 g) was used for the extraction of the enzyme with 2 mL of extraction buffer. The enzyme activity was measured at 340 nm. For the assessment of GR activity, 6.2 mM^−1^ cm^−1^ was used as the molar absorptivity constant, and the result was represented as EU mg^−1^ protein min^−1^.

##### Ascorbate peroxidase (EC 1.11.1.11)

Nakano and Asada^48^ a protocol was employed to assess the Ascorbate peroxidase **(**APX) activity.

A fresh sample (0.3 g) was used for enzyme extraction. The reaction was performed in 2 mL of reaction buffer (50 mM phosphate buffer), 1% (w/v) polyvinylpyrrolidone (PVP), 1% (w/v) Triton X−100, and EDTA (0.3 mM) were added. The enzyme activity was checked at 290 nm, calculated using the extinction coefficient (e) 2.8 mM^−1^ cm^−1^, and expressed as EU/mg^−1^ protein min^−1^.

#### Total sugar estimation

Total soluble sugar was estimated in the callus and shoot as per the anthrone method with some modifications.^49^ 300  mg of dried callus and shoot were made into powder with liquid nitrogen, and 80% ice-cold ethanol was used for the isolation. The mixture was centrifuged at 5000 rpm at 4 °C for 5 min. 3 mL of freshly made anthrone reagent was mixed with 1 mL of supernatant, and the mixture was incubated for 10 min at 100 °C in a water bath. The reaction was stopped by cooling on ice and kept at room temperature. The absorbance was measured with a spectrophotometer at 620 nm (UV–visible spectrophotometer; Shimadzu). The standard curve for sugar calculation was established using the glucose concentrations of 10, 20, 30, and 40 µg/mL. The total sugar content in the sample was determined using the linear equation *y* = 0.0064*x* + 0.026 (*R*² = 0.993) and expressed in µg g^−1^ DW.

### Assessment of genetic fidelity

#### Nuclei extraction protocol

Pure nuclei were extracted from callus and shoot with minor modifications in the protocol as developed by Sadhu et al.^50^ The MB01 buffer consists of various chemical components (20 mM MOPS; 0.7 mM spermine; 25 mM Na_2_EDTA; 4HCl; 20 mM NaCl; 80 mM KCl; 0.5% (v/v) β-mercaptoethanol; 1% (w/v) PVP; and 0.2% (v/v) Triton X-100) was employed for the isolation of nuclei.^50^ Three samples were taken from each treatment of callus and shoot as replicates to extract nuclei. The young and fresh samples (weighing 30 mg shoot and 40 mg callus) were cut into small pieces with a razor (shoot) and randomly chopped 5 to 6 times (callus) in a cold isolation buffer (700 µL). The crude nuclei were passed through a double layer of nylon mesh (pore size 20 µm) to remove the debris. The extracted nuclei in the buffer were stained with 50 µg/mL of PI dye (propidium iodide) and kept on ice for 8 min.

#### Flow cytometry analysis

The genome size (2C DNA content) was calculated for treated callus and shoot samples, following the methodology outlined by Dolezel et al.^51^ To ensure accurate results, the flow rate of the capillary was adjusted to a minimum of 0.12 µL/s. The calculation of genome size, *Solanum lycopersicum* (2C = 1.96 pg), was used, which was provided by Dr. Jaroslav Dolezel from the Laboratory of Molecular Cytogenetics and Cytometry at the Institute of Experimental Botany in the Czech Republic. A total of 5000 nuclei stained with propidium iodide were analyzed using the FACS Muse cell analyzer (Sigma, USA). The histograms produced during the scanning of the nuclei were used for the analysis and further genome size estimation according to the following formula:

$$\;\begin{array}{l} 2C\;\mathrm{DNA}\;\mathrm{content}\;\mathrm{of}\;\mathrm{Reseda}\\ \;=\frac{\mathrm{Florescence}\;\mathrm{mean}\;\mathrm{intensity}\;\mathrm{of}\;\mathrm{Reseda}\;}{\mathrm{Floresence}\;\mathrm{mean}\;\mathrm{intensity}\;\mathrm{of}\;\mathrm{standard}}\times 2C\;\mathrm{DNA}\;\mathrm{content}\;\mathrm{of}\;\mathrm{standard}. \end{array}\;$$

### Extraction of total phenolic content (TPC) and flavonoid content (TFC)

The phenolic compounds were extracted from dried callus and shoots in methanol. One gram of powder from callus and shoot was added to 20 mL of methanol and kept overnight on a shaker at room temperature. Thereafter, the mixture was filtered through Whatman filter paper. The samples were dried under reduced pressure and finally prepared in methanol for the determination of TPC and TFC.

#### Total Phenolic content (TPC) estimation

Spectrophotometric measurements of total phenolics were made using the Folin–Ciocalteu reagent.^52^ After mixing the sample (100 µL) with water (900 µL), 75 µL of Folin–Ciocalteu reagent was added. The incubation process was performed in complete darkness. The mixture was thoroughly mixed with 125 µL of 20% Na_2_CO_3_. With a UV–visible spectrophotometer (Shimadzu, USA), the absorbance of the blue complex was determined at 725 nm. The standard curve for TPC calculation was prepared using the various concentrations of 25, 50, 100, 150, and 200 µg/mL (gallic acid as a standard). The TPC was calculated in the sample using the linear equation *y* = 0.0263*x* + 0.020 (*R*^2^ = 0.998) and represented as µg/g GAE.

#### Total flavonoid content (TFC) estimation

Using the colorimetric approach as previously reported by Zhishen et al.^52^, the total flavonoid content was measured. The reaction was carried out with 200 µL of plant extract, 1000 µL of water, and 38 µL of 5% NaNO₂ combined in a glass tube to set up the reaction. Following a 10-min incubation period, 75 µL of a 10% AlCl₃ solution was added and left for 5 min. The reaction mixture mentioned above was supplemented with 250 µL of 1 M NaOH. A UV–visible spectrophotometer (Shimadzu, USA) was used to determine the absorbance at 510 nm. The quercetin (QE) was taken as 40, 80, 120, 160, and 200 µg/mL for standard curve preparation, and the content in the sample was calculated using the linear equation *y* = 0.002*x *+ 0.002 (*R*² = 0.996) in µg/g QE.

### Statistical analysis

The experiment was carried out in replicates (*n* = 3) to validate the consistency of the results extracted from the individual treatment carried out on callus and shoot cultures. The statistical analysis was carried out using software (SPSS, version 11, SPSS Inc., Chicago, USA) and one-way analysis of variance (ANOVA). Duncan's test was performed to compare the means at 5% level (the results are represented as the means ± SE).

## Results

### Biosynthesis of ZnO-NPs and characterization

**Figure 1.**
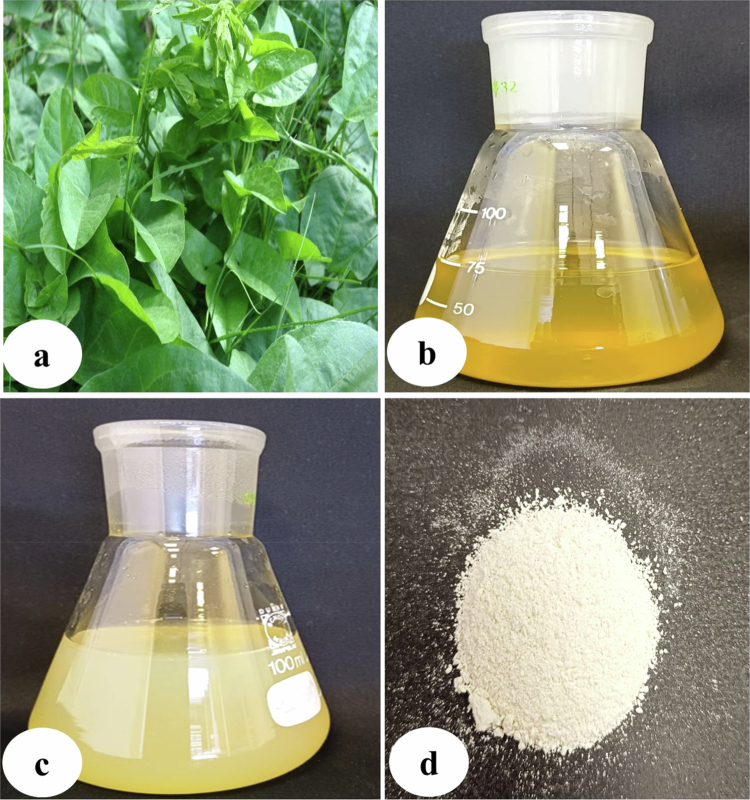
Synthesis of zinc oxide nanoparticles using aqueous extract of *Convolvulus arvensis.* (a) Plant of *Convolvulus arvensis*; (b) aqueous leaf extract of *C. arvensis*; (c) synthesized zinc oxide nanoparticles in crude form; and (d) calcined zinc oxide nanoparticles in powder form.

**Figure 2.**
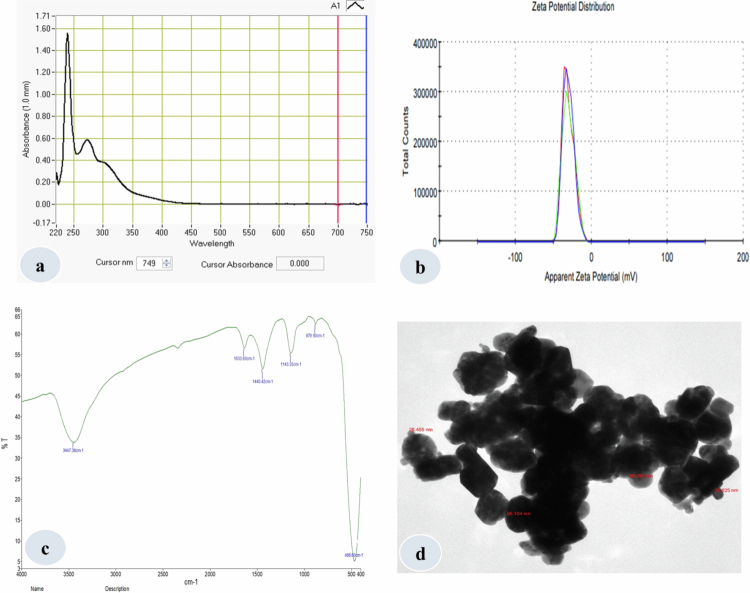
Results of ZnO-NPs characterization using different techniques (a) UV–visible absorption spectra of ZnO-NPs; (b) zeta potential; (c) FT-IR; and (d) TEM image of ZnO-NPs.

The biogenic synthesis of ZnO-NPs was carried out using an aqueous extract of *C. arvensis,* which was identified by the internal transcribed spacer sequence of rDNA (GenBank accession number: KJ021876). The synthesis of ZnO-NPs was confirmed by the color change of the zinc nitrate solution upon mixing an extract of *C. arvensis* at 60 °C (Figure 1). Further, different techniques were employed, including UV–vis spectroscopy, zeta potential, Fourier transform infrared (FT-IR) spectroscopy, and transmission electron microscopy (TEM) for characterization (Figure 2), which were carried out at the Department of Chemistry, King Saud University. The biogenic nanoparticles synthesis was checked using a UV–vis spectrophotometer (NanoDrop 8000 spectrophotometer) by taking the spectra. The clear peak of UV spectra was recorded at 280 nm (ZnO-NPs), which confirms the nanoscale size of nanoparticles with the aqueous extract of *C. arvensis*. FT-IR spectra were recorded from 400–4000 cm^−1^ wavelength range. The various absorption bands detected by FT-IR at different wavelengths were 3447.36 cm^−1^, 1633.60 cm^−1^, 1440.42 cm^−1^, 1143.35 cm^−1^, 879.93 cm^−1^, and 466.60 cm^−1^ for ZnO-NPs. The absorption band identified at 3447.36 cm^−1^ showed a broad and strong peak that was produced from the stretching and vibration of an O–H functional group. The peak identified at 1633 cm^−1^ was verified for the compound having a functional group (C = C stretching). Similarly, the band recorded at 1440.42 cm^−1^ was found with a functional group with O–H bending. The transmission electron microscopy (TEM) exhibited the size of ZnO-NPs ranged from 26.455 nm to 98.104 nm, which reveals the spherical synthesis of ZnO-NPs with an approximate uniform size distribution. The zeta potential (−30.6 mV) recorded for ZnO-NPs with a conductivity of 0.141 mS/cm shows its stability. Thus, the above findings reveal the effective and reliable synthesis of ZnO-NPs through a biogenic route using *C. arvensis* extract*.*

### Callus induction and shoot production using plant growth regulators (PGRs)

**Figure 3.**
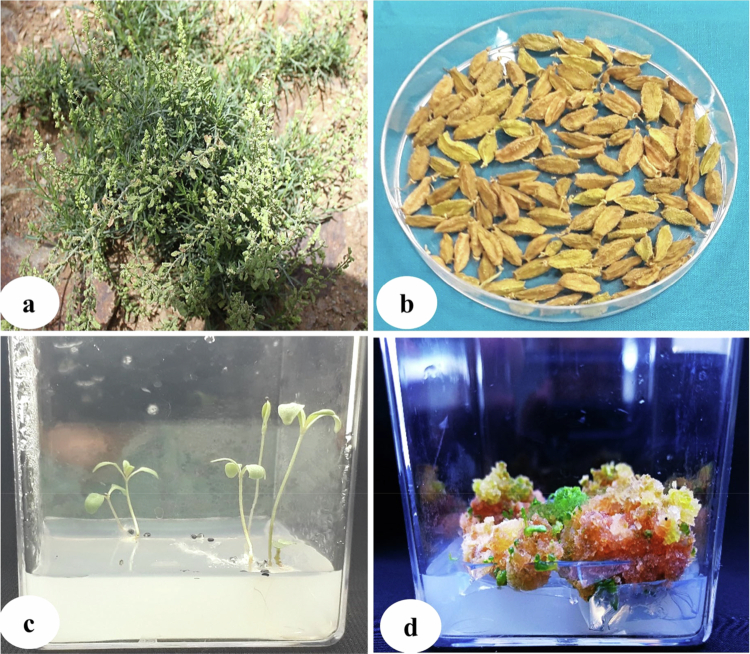
(a) Plant collected from wild conditions; (b) collected fruits with seeds; (c) seed germination on simple agar (0.8%); and (d) callus produced from cotyledons.

**Figure 4.**
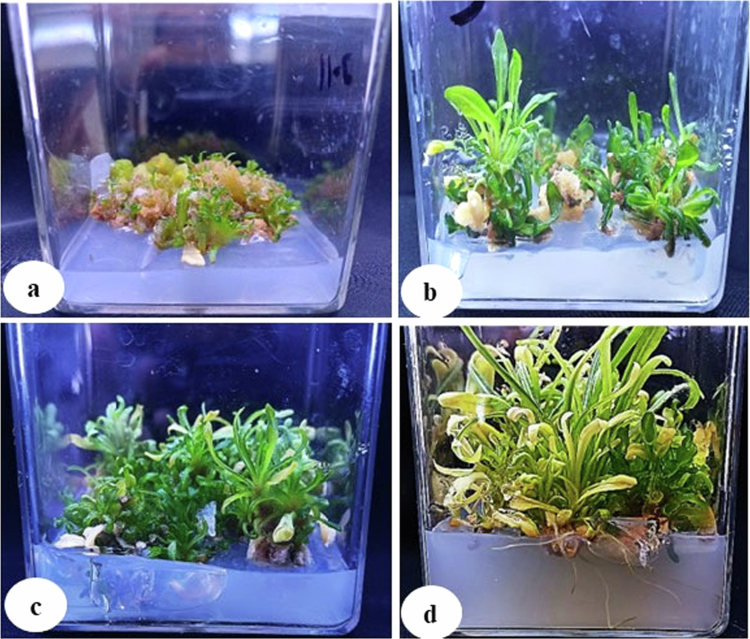
Indirect organogenesis (from the callus); (a) shoot induction with BAP (0.4 mg/L); (b) shoot regeneration on MS media without PGR; (c) shoot regeneration with BAP (0.4 mg/L); and (d) root induction (0.5 mg/L IBA).

The collected plant and seeds of *R. lutea* were identified by a botanist as well as using the molecular markers (internal transcribed spacer sequence of rDNA: accession number-KF805108) (Figure 3), after which further seeds were cryopreserved. The sterilized seeds were germinated on 0.8% agar to get cotyledons (Figure 3). The cotyledons of germinated seeds were made into small pieces of size (10 mm × 10 mm squares) and transferred aseptically to MS medium supplemented with PGRs (0.3 mg/L 2,4-D with 0.1 mg/L 6-benzylaminopurine (BAP)) (Figure 3). The callus was initiated easily after 2 weeks of explant culture (cotyledons) on MS medium. The bulk callus tissue (after 4 weeks of culture) was subsequently transferred to MS media for ZnO-NP treatment. The callus was obtained in bulk after 4 weeks of culture, which was used further for the treatment with biogenic ZnO-NPs. The shoots were induced from the callus using different concentrations of BAP (0.1, 0.2, 0.3, and 0.4 mg/L) supplemented on MS medium. The induction of shoots was initiated after two weeks of callus culture on MS medium supplemented with BAP. Among the various concentrations of BAP, the best result was found with supplementation of 0.4  mg/L on MS medium (Figure 4a), which was further used on MS media for multiplication of shoots, and also multiple shoots were produced without PGRs (Figure 4b,c). Multiple shoots were produced in bulk from induced shoots after four weeks of culture. The regenerated shoots were subcultured on root induction media added with different concentrations of IBA (0.1, 0.2, 0.3, 0.4, and 0.5  mg/L), and the best result was recorded at 0.5 mg/L IBA (Figure 4d)**.**

### Assessment of ZnO-NPs impact on callus and shoot growth

**Figure 5.**
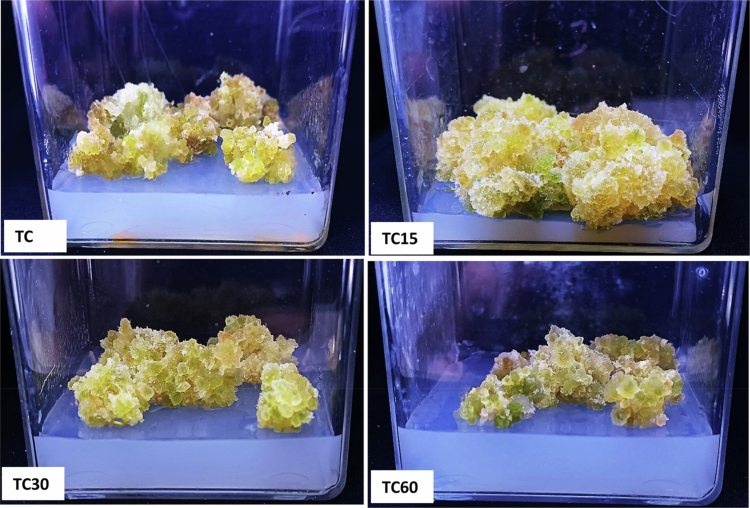
Effect of ZnO-NPs on callus growth and morphology; TC = control; TC15 = callus treated with 15 mg/L of ZnO-NPs; TC30 = callus treated with 30 mg/L of ZnO-NPs; and TC60 = callus treated with 60 mg/L of ZnO-NPs.

**Figure 6.**
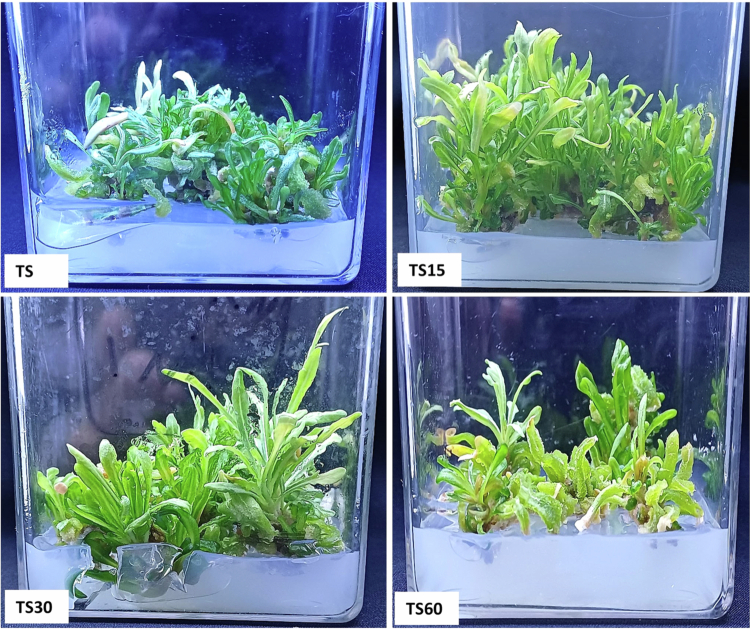
Effect of ZnO-NPs on shoot growth and morphological parameters; TS = control; TS15 = shoot treated with 15 mg/L of ZnO-NPs; TS30 = shoot treated with 30 mg/L of ZnO-NPs; and TS60 = shoot treated with 60 mg/L of ZnO-NPs.

**Figure 7.**
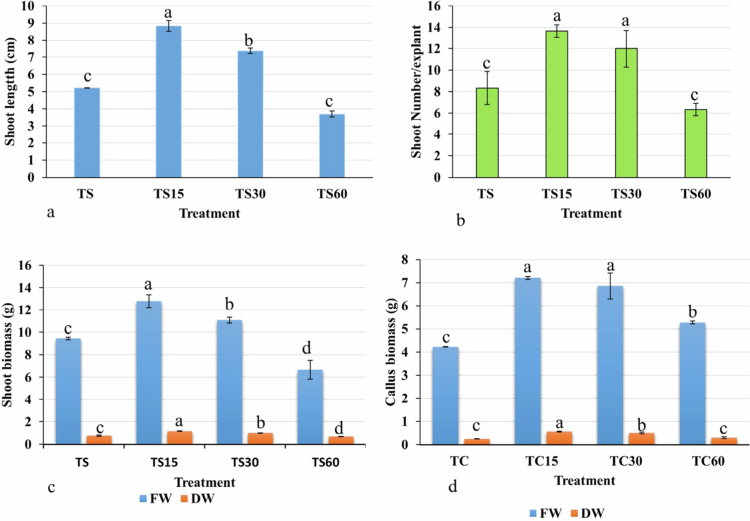
Effect of ZnO-NPs on morphological traits of callus and shoot of *Reseda lutea*; (a) shoot length; (b) shoot number; (c) shoot fresh and dry weight; and (d) callus fresh and dry weight. Different letters represent the significant results (*p* ≤ 0.05).

Figure 5 shows the response of callus with variation in morphology at different concentrations of ZnO-NPs supplemented on MS medium. The callus treatment with ZnO-NPs at concentrations of 15 mg/L and 30 mg/L, showed a higher fresh weight by 70.77% and 62.63% respectively, compared to the control significantly (Figure 7). The callus exhibited a browning color with 60 mg/L ZnO-NPs; however, it showed a positive response in terms of fresh weight by 24.82% than the control. However, a non-significant result was observed in dry weight with the same amount of ZnO-NPs (60 mg/L) than the control. In contrast to callus, the biomass of the shoot was reduced by 29.39% with 60 mg/L of ZnO-NPs treatment than the control (Figure 6). The shoot length, biomass, and shoot number were enhanced significantly at 15 and 30 mg/L of ZnO-NPs treatment than the control group (Figure 7). The fresh weight accumulation in shoots treated with 15 mg/L of ZnO-NPs was improved by 35.38%, which was lower than the response monitored in the callus.

### Effect of ZnO-NPs on chlorophyll content in the shoot

**Figure 8.**
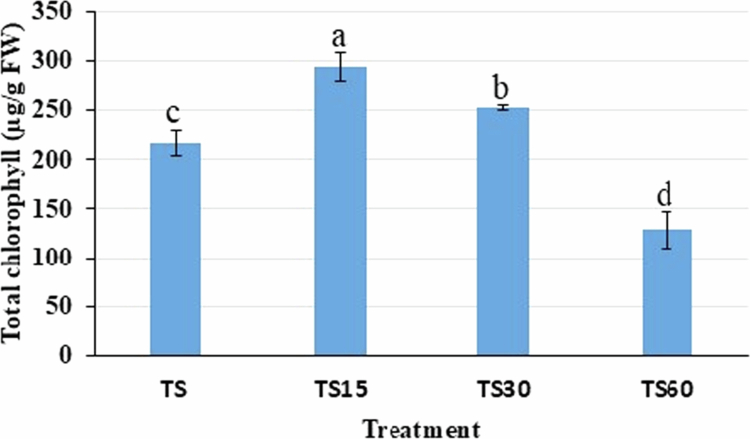
Total chlorophyll content (a + b) in the shoot of *Reseda lutea* under the treatment of ZnO-NPs.

Among the shoot treatments, the total chlorophyll content was enhanced by 37.85% and 16.78% significantly in the shoots treated with 15 and 30 mg/L ZnO-NPs, as compared to the control (Figure 8). However, it was decreased by 40.78% with 60 mg/L ZnO-NP treatment than the control.

Treatment includes, TS = shoot control; TS15 = shoot treated with 15 mg/L ZnO-NPs; TS30 = shoot treated with 30 mg/L ZnO-NPs; and TS60 = shoot treated with 60 mg/L ZnO-NPs. Different letters represent significant results among the treatments and the control (*p* ≤ 0.05).

### Effect of ZnO-NPs on total crude protein accumulation

Protein plays an important role in various physiological activities of the plant, such as growth, development, and stress response. The shoot had a higher total protein content than the callus in the control as well as in the treatments under ZnONPs. Among the callus and shoot treatments, protein content was enhanced in the callus by 36.4% and 14.85%, whereas in the shoot, it was increased by 10.67% and 26.24% with 15 mg/L and 30 mg/L ZnO-NPs treatment compared to the control, respectively (Table 1). The protein content sharply decreased both in callus and shoot with 60 mg/L ZnO-NPs than the control.

### Impact of ZnO-NPs on total sugar content

Sugar regulates growth and development and also helps in the osmotic balance of the cell. The total sugar content varied between callus and shoot with different concentrations of ZnO-NPs treatment, and it was higher in the shoot than in the callus (Table 1). Among the various treatments, the content of total sugar was found to be higher in the treated shoots and calluses than in the control. The sugar content in callus and shoot of *R. lutea* treated with 15, 30, and 60  mg/L ZnO-NPs was enhanced by 22.6%, 6.7% and 39.9%, whereas in the shoot by 78.6%, 70.6% and 28.3%, respectively. The highest sugar content was found in the shoot and callus treated with 60 mg/L and 15  mg/L of ZnO-NPs, than the control, respectively.

**Table 1.** Total crude protein, sugar, and polyphenol content in the callus and the shoot of *Reseda lutea* under ZnO-NPs treatment.

**Treatment**	**Total protein (µg/g FW)**	**Total sugar (µg/g DW)**	**TPC (µg/g GAE)**	**TFC (µg/g QE)**
TC	3080 ± 0.04b	150 ± 2.70c	610.784 ± 12.81b	370.588 ± 17.64c
TC15	4210 ± 0.14a	183.90 ± 3.19b	652.156 ± 12.72b	421.568 ± 23.52c
TC30	3540 ± 0.38b	196.35 ± 12.13ab	659.607 ± 5.33b	504.902 ± 47.27b
TC60	2820 ± 0.25c	209.89 ± 15.65a	705.490 ± 43.13a	586.274 ± 17.97a
TS	13730 ± 1b	207.81 ± 4.68c	1145.098 ± 14.80b	733.333 ± 3.96c
TS15	17330 ± 0.92a	371.35 ± 14.76a	1143.137 ± 27.16b	784.313 ± 22.27b
TS30	15200 ± 1.2b	354.68 ± 16.53a	1198.039 ± 23.77a	827.45 ± 20.65a
TS60	10000 ± 0.8c	266.67 ± 26.43b	1139.21 ± 6.79b	696.07 ± 17.97d

Treatment includes, TC = callus control; TC15 = callus treated with 15 mg/L ZnO-NPs; TC30 = callus treated with 30 mg/L ZnO-NPs; TC60 = callus treated with 60 mg/L ZnO-NPs; TS = shoot control; TS15 =​​​​​​ shoot treated with 15 mg/L ZnO-NPs; TS30 = shoot treated with 30 mg/L ZnO-NPs; TS60 = shoot treated with 60 mg/L ZnO-NPs; different alphabets denote the significant results among treatment and control (*p *≤ 0.05).

### Modulation of total phenolic and flavonoid content (TPC and TFC) by ZnO-NPs treatment

The phenolic compounds play an important role in plant defense mechanisms and in protecting plants from various abiotic stresses. In the present study, the total phenolic content in the callus increased by 6.7%, 1.1%, and 1.5% with 15, 30, and 60 mg/L of ZnO-NPs treatment, respectively, while a notable positive response was observed in the shoot at 30 mg/L (4.6%) (Table 1). Similarly, the total flavonoid content increased by 13.7%, 19.76%, and 58.20% in the callus, whereas it increased by 6.9% and 12.8% in the shoot with 15 and 30 mg/L ZnO-NPs treatments, respectively. However, both TPC and TFC were decreased in the shoots with 60 mg/L of ZnO-NPs treatment. More accumulation of TPC and TFC was detected in the shoots than in the callus with ZnO-NPs treatment. This variation in polyphenolic content in callus and shoots of *R. lutea* following ZnO-NP treatment might be due to the equilibrium between nutritional stimulus and stress-activated defense.

### Impact of ZnO-NPs on TBARS content

Thiobarbituric acid reactive substances (TBARS) are generally determined as a marker of lipid peroxidation and oxidative stress in plants. They are produced because of the breakdown of polyunsaturated fatty acids present in the cell membranes under various types of stress conditions. The content of TBAR was found to be highest in the callus (37.25%) and shoot (63.8%) with 60 mg/L ZnO-NP treatment (Table 2). The lowest content of TBAR accumulation was found to be with 15 mg/L ZnO-NPs treatment compared to the control, in both shoot and callus. More accumulation of TBARS was recorded in the shoot compared to the callus with ZnO-NPs treatment. There was no significant variation found in TBAR content between the control and regeneration stages (callus and shoot) treated with 15 mg/L ZnO-NPs. The low content of TBARS produced in the callus and shoot with low concentration of ZnO-NPs (15 mg/L) treatment showed the protective role in membrane peroxidation.

### Effect of ZnO-NPs on proline accumulation

Proline is an amino acid and osmolytes that protect the plant under abiotic stress conditions. The content of proline increased in all the treated calluses as well as shoots in a dose-dependent manner. A non-significant result was found in the accumulation of proline content in both regeneration stages (callus and shoot) treated with 15 mg/L of ZnO-NPs. The proline content was found to be higher in the shoot than in the callus with ZnO-NPs treatment. The highest accumulation of proline (190.8% and 179.8%) was observed with 60 mg/L of ZnO-NPs treatment in the callus and shoot, than the control, respectively. Thus, accumulation of proline at various concentrations of ZnO-NPs treatment indicated the oxidative stress, resulting in the generation of ROS.

### Antioxidant defense response under ZnO-NPs treatment

The antioxidant enzymes play a potential function in defending plants from injury as triggered by reactive oxygen species (ROS), which are destructive byproducts of cellular metabolism under abiotic stress. A non-significant result was found in enzyme activities, including GR and APX in callus and shoot with 15 and 30 mg/L of ZnO-NPs as compared to the control (Table 2). However, the activity of antioxidant enzymes (APX and SOD) was enhanced in both callus and shoot treated with 30 and 60 mg/L of ZnO-NPs compared to the control. The activities of the enzymes, including GR, SOD, and APX, increased with 60 mg/L of ZnO-NPs by 31.6%, 196%, and 270% in callus, whereas in shoots by 119%, 162%, and 313% relative to the control, respectively. However, a non-significant result was observed in the activities of APX and GR in callus and shoot with 15 and 30  mg/L of ZnO-NPs than in the control. However, calluses exhibited SOD activity in the same way both in the control as well as in the treatment with 15  mg/L of ZnO-NPs, after which it enhanced in a dose-dependent manner. The activity of GR trend was found to be different from the SOD and APX activities. However, the SOD and APX activity was found to be greater in the callus with 60 mg/L ZnO-NPs than in the shoot.

**Table 2.** Antioxidant system response in the callus and the shoot of *Reseda lutea* under treatment of ZnO-NPs.

**Treatment**	**Proline (µg/g FW)**	**TBAR content (nM/g FW)**	**GR** **(EU/mg protein/min)**	**SOD** **(EU/mg protein/min)**	**APX** **(EU/mg protein/min)**
TC	37.55 ± 2.54c	0.038 ± 0.00b	0.182 ± 0.067a	0.775 ± 0.116c	0.339 ± 0.049b
TC15	47.38 ± 3.40c	0.039 ± 0.01b	0.163 ± 0.04a	0.776 ± 0.03c	0.395 ± 0.09b
TC30	82.0 ± 5.77b	0.050 ± 0.01b	0.173 ± 0.10a	1.21 ± 0.07b	0.481 ± 0.17b
TC60	109 ± 11.09a	0.179 ± 0.04a	0.24 ± 0.03a	2.29 ± 0.15a	1.25 ± 0.16a
TS	42.0 ± 7.26c	0.031 ± 0.00c	0.156 ± 0.03b	0.482 ± 0.02c	0.190 ± 0.021b
TS15	52.0 ± 6.0c	0.049 ± 0.01bc	0.156 ± 0.03b	0.315 ± 0.05c	0.200 ± 0.05b
TS30	67 ± 2.88b	0.061 ± 0.061b	0.199 ± 0.08b	1.0 ± 0.07b	0.272 ± 0.09b
TS60	117 ± 8.85a	0.229 ± 0.021a	0.343 ± 0.16a	1.26 ± 0.12a	0.786 ± 0.30a

Different letters denote the significant results between the treatments and the control (*p* ≤ 0.05).

### Determination of genome size in callus and shoots under ZnO-NPs treatment

**Figure 9.**
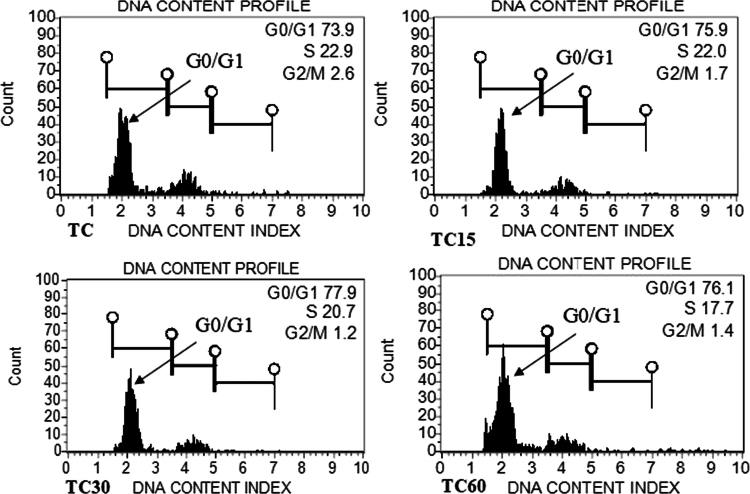
Histogram peak from flow cytometry analysis depicts fluorescence intensity distribution of the cell (callus) treated with ZnO-NPS: TC = control callus; TC15 = callus treated with 15 mg/L of ZnO-NPs; TC30 = callus treated with 30 mg/L of ZnO-NPs; and TC60 = callus treated with 60 mg/L of ZnO-NPs.

**Figure 10.**
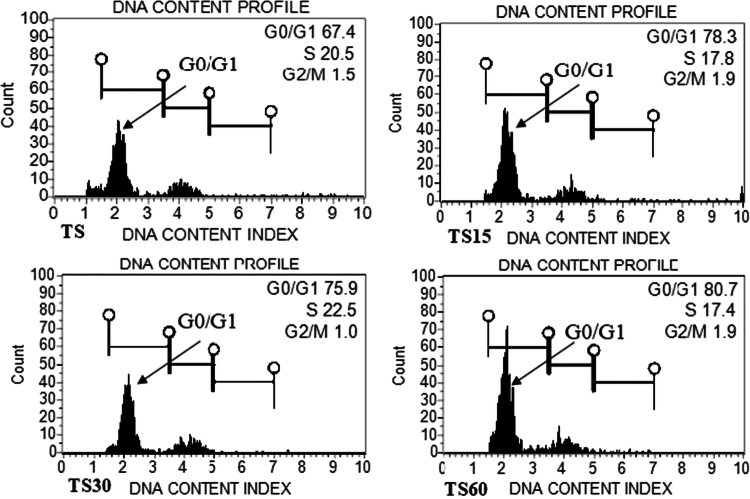
The flow cytometry-generated histogram peak shows the cell's distribution of fluorescence intensity: (TS = shoot control; TS15 = shoot treatment with 15 mg/L of ZnO-NPs; TS30 = shoot treated with 30 mg/L of ZnO-NPs; and TS60 = shoot treated with 60 mg/L of ZnO-NPs.

Flow cytometry is commonly used in plant taxonomy and breeding programs to assess genetic stability and detect polyploidy levels. The genome size (2C DNA content) ranged from 1.91 to 1.92 in callus, which was calculated from the fluorescence intensity of G0/G1 phase, whereas it was recorded from 1.91 to 1.93  pg in shoot as compared to controls (1.91 pg), respectively. A non-significant minor variation was detected in callus and shoot treated with 60 mg/L of ZnO-NPs, relative to the control. However, the addition of low concentrations of ZnO-NPs, including 15 and 30  mg/L in MS medium, did not affect the genome size stability of both callus and shoot, which indicates their proper growth and development. The histogram shown in Figures 9 and 10 confirms the genetic stability of the callus and the shoot at various concentrations of ZnO-NP treatment.

**Figure 11.**
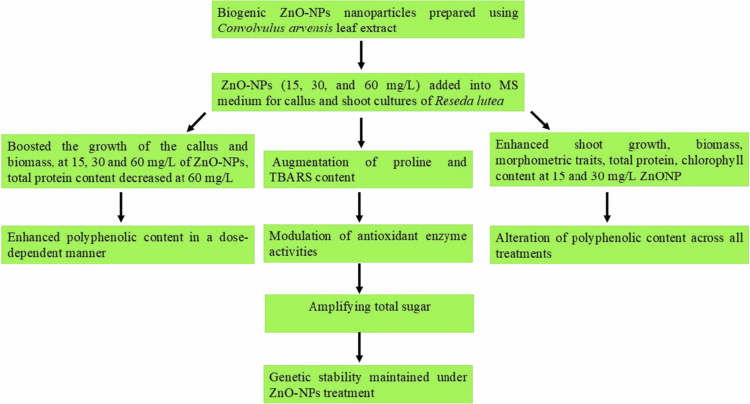
Effect of biogenic ZnO-NPs on regeneration stages (callus and shoot) of *Reseda lutea.*

## Discussion

Zinc oxide nanoparticles (ZnO-NPs) play a significant role in the modulation of physical, chemical, and biological attributes of the plants. Phytosynthesized zinc oxide nanoparticles have many advantages over chemically produced ones. Moreover, biogenic ZnO-NPs can have a positive impact on plant productivity and other growth indices, and the green synthesis method is considered environmentally safe.^53^ The present study focused on the callus and regenerated shoots of *R. lutea* under the treatment of biogenic zinc oxide nanoparticles, which were prepared using the aqueous extract of *C. arvensis*. The callus of *R. lutea* was initiated on MS media from the cotyledons of germinated seeds with different PGRs combinations of 2,4-D with BAP, which were supplemented with MS medium to obtain the healthy callus. The fully developed callus (two-month-old), free from browning and necrosis, compact, uniform in color (light green), and friable in texture, was obtained in bulk with a combination of 0.3  mg/L 2,4-D and 0.1  mg/L BAP after screening of various combinations of PGRs (Figure 3d).

Depending on the dose and length of exposure, ZnO-NPs have different effects on different plant organs.^36^ The developed callus of *R. lutea* was treated with various concentrations of biogenic ZnO-NPs, viz., 15, 30, and 60 mg/L, with a reduced concentration of 2,4-D (0.1 mg/L) compared to the 0.3 mg/L concentration previously used for bulk callus production. All concentrations of ZnO-NPs (15, 30, and 60  mg/L) showed a positive effect on callus growth and biomass (Figure 4b–d); however, their response levels varied. The low concentrations of ZnO-NPs (15 mg/L) showed better results on biomass accumulation in *R. lutea,* which might be due to the augmentation of different types of enzyme activities. NPs play a significant role in the induction of callus and shoots, and their positive effect on growth has been observed in various crops under the application of optimum concentrations.^54^^,^^55^ A high concentration of zinc nanoparticles can stop plant growth, whereas suitable concentrations can stimulate the growth and development of the plant.^56^ ZnO-NPs induced the callus production and shoot production in *Brassica napus*, *Brassica nigra*, *Panicum virgatum,* and *Delonix elata* cultured on MS media.^36^^,^^57^^-^^59^ The calluses induced from the tips of the banana shoot, the number of shoots per callus increased with an increase in ZnO-NPs on MS medium.^60^

In a comparative study of the callus and shoot of *R. lutea* under ZnO-NPs treatment, a more pronounced effect was seen on the callus compared to the shoot treatment. Among all the concentrations of nanoparticles applied on the shoot of *R. lutea*, a low concentration of ZnO-NPs (15 mg/L) proved to have a promoting effect as recorded from shoot number, shoot length, and shoot biomass (fresh and dry weight). However, a high concentration of ZnO-NPs (60 mg/L) negatively affected shoot biomass (fresh and dry weight), shoot length, and shoot number, as a high dose of nanoparticles might induce oxidative stress, which affects the growth parameters and eventually decreases the biomass. ZnO-NPs enter the plant tissues through absorption and translocation, accumulated over time in intracellular and intercellular spaces, and cause physiological inhibition, nutritional imbalance, and disruption of photosynthesis in plants.^61^ The promoting effect of ZnO-NPs on callus and shoots of *R. lutea* was supported by *in vitro* culture of olive, *Ochradenus arabicus*, *Eriobotrya japonica*, and *Juniperus procera*.^20^^,^^21^^,^^62^^,^^63^

Zinc nanoparticles (ZnO-NPs) modulate the biochemical profile of the plants, increasing the synthesis and accumulation of various biomolecules that play a significant role in plant growth and development. ^33^^,^^64^ This might be due to the slow release of ZnO-NPs into Zn^+2^, ensuring prolonged availability than the bulk zinc salts. The easy absorption of nanosized zinc by plant impacts various physiological activities of the plant.^56^ The different concentrations of ZnO-NPs improved the soluble sugars, phenolic compounds, and protein content in the callus of *R. lutea*; however, high concentrations of ZnO-NPs (60 mg/L) negatively affected shoot growth development and various growth parameters (Figure 6). Moreover, the content of total protein, TPC, and TFC was decreased compared to the control. The zinc nanoparticles have been found to directly enhance the content of metabolites and osmoprotectants in plants, including glycine, amino acids, proline, sugars, and betaine.^65^ These compounds play a potential role in dealing with stress by stabilizing cellular structures, protecting against oxidative damage, and maintaining osmotic balance.^66^ The high concentrations of ZnO-NPs produce free radicals, which ultimately cause oxidative stress and lipid peroxidation. TBARS are produced from lipid peroxidation of the cell membrane in plants under abiotic stress. The high concentrations of zinc nanoparticles (60 mg/L) employed in the MS medium, caused more TBARS accumulation in both callus and the shoot of *R. lutea*, indicating more lipid peroxidation of the cell membrane. The present results on *R. lutea* are reinforced by the results of Kumari et al.,^67^ where the accumulation of TBARS was found to be in a dose-dependent manner in *Allium cepa* under ZnO-NP treatment.

The stress marker proline is an active osmolyte, an antioxidative defense molecule, and a metal chelator that protects the plant cells from abiotic and biotic stresses. The content of proline was found to be more in the shoots of *R. lutea* than in the callus with 60 mg/L of ZnO-NPs, and it was found to be a function of the dose. Proline protects the cell membrane structure under various stress conditions, such as drought, salinity, and temperature stress^68^ and stabilizes membranes, maintains osmotic balance, and scavenges ROS during stress. In another study, it was found that high levels of proline denote the plant's defensive mechanism against oxidative damage caused by ZnO-NPs.^29^ The proline level was enhanced in the callus and shoots of the banana upon exposure to nano-zinc and ZnO-NPs.^60^ Similarly, upon the addition of high concentrations of ZnO-NPs *in vitro* culture shoots of *Ochradenus arabicus* as well as plants grown in a growth chamber (*Echinops macrochaetus*), showed elevated content of proline.^29^^,^^43^ The crude protein is a gauge of the plant's basic metabolic processes. It denotes the nitrogen-based compounds that the plant requires for growth, development, and self-defense. At optimum concentration of ZnO-NPs, which have been described to improve protein synthesis by improving nutrient uptake, stimulating enzymatic activity, and promoting metabolic routes important for amino acid and protein production. The low doses of ZnO-NPs (15 and 30 mg/L) improved the chlorophyll and protein content in the shoots of *R. lutea* by improving the stress tolerance (Table 1 and Figure 8). The application of biogenic ZnO-NPs on *B. napus* improved the protein synthesis and transcriptome profiling positively, therefore inducing the growth and development of the plant.^69^ However, a high dose of ZnO-NPs (60 mg/L) showed a toxic effect on the shoots of *R. lutea,* which might be caused by the production of free radicals, leading to disturb the physiological activities of the cell. More accumulation of chlorophyll could be correlated with a high content of protein synthesis, which indicates a sign of healthy growth and effective metabolism in the shoot of *R. lutea*. The ZnO-NPs improved the chlorophyll content in crops such as wheat and the protein content in green gram and tea.^30^^,^^31^^,^^70^

The high concentration of zinc nanoparticles caused toxicity in plant cells and eventually inhibited growth and development. Hence, plants adopt a diverse approach in the form of antioxidant defense to scavenge reactive oxygen species (ROS).^71^ The antioxidants present in plants can ameliorate the toxicity of H_2_O_2_ by changing it into water and oxygen.^72^ The low concentration of ZnO-NPs (15mg/L) increased the growth of the callus and shoot in *R. lutea* (Figures 5 and 6); however, the activity of APX and SOD was found to be higher with 30 and 60mg/L of ZnO-NPs treatment, which was opposite to the GR activity trend in the callus at 30 mg/L (nonsignificant). The content of the enzyme depends on the physiological activity of the plant cells and also the ROS level. In an earlier study, it was found that ZnO-NPs increased the activity of the antioxidant enzymes, superoxide dismutase and catalase in the callus of *Solanum lycopersicum.*^73^ Similarly, various concentrations of ZnO-NPs increased the free radical levels and lipid peroxidation in the callus of *Linum usitatissimum.*^54^ Our results showed that treatment with a high concentration of ZnO-NP (60 mg/L) increased the activity of antioxidant enzymes (SOD, GR, and APX) in the callus and shoots of *R. lutea* (Table 2). These enzymes help in the effective elimination of free radicals, which are harmful byproducts of cellular metabolism that build up during stressful situations. In the antioxidant defense system, the first line of defense is SOD, which changes O^2–^ to H_2_O_2_ less toxically.^74^ At different treatment doses of ZnO-NPs on *Hordeum vulgare*, the levels of proline, catalase (CAT), SOD, and APX were increased.^75^

The small size of ZnO-NPs (size: 26.48−98.10nm), which were synthesized using the leaf extract of *C. arvensis* improved the growth of callus and shoot, enzymatic activities, chlorophyll content, and total sugar in *R. lutea*. Sugar accumulation in plants plays a vital role in both stress tolerance and the enhancement of therapeutic properties. Sugars act as a compatible solute, helping maintain cell turgor and protecting proteins and membranes under environmental stresses, such as salinity, drought, and cold. Moreover, sugars provide carbon skeletons to produce secondary metabolites, such as flavonoids, alkaloids, and terpenoids, which are often important medicinal compounds. Sugar molecules also act as signaling molecules to induce the biosynthesis of secondary metabolites, which provide a defense response to biotic and abiotic stresses, thereby supporting the survival of the plant.^76-78^ It has been demonstrated that ZnO-NPs increase the accumulation of transport sugar (sucrose) and photosynthetic products in plants, which promotes the transfer of carbon and energy to sink tissues.^79^ There was a little variation found in the total sugar content in the callus and shoot of *R. lutea* with ZnO-NP treatment. However, more sugar accumulated in the shoots with ZnO-NPs treatment, and it could be correlated with greater accumulation of chlorophyll (Table 1 and Figure 8). The sugar produced in *R. lutea* under the application of ZnO-NPs might act as a signaling molecule, which helps increase the accumulation of TPC and TFC. Our result is supported by the result of Awan et al.,^80^ where the application of ZnO-NPs enhanced the content of total sugar. Similarly, in another study, different concentrations of zinc oxide NPs enhanced the soluble sugar content in fresh tea leaves.^30^

Nanoparticles have been recognized as novel elicitors that can trigger antioxidant defense mechanisms.^81^ However, the effectiveness of induction can vary depending on many factors, such as the concentration of nanoparticles, culture conditions, and treatment duration. In our study, the accumulation of total phenolic and flavonoid content in the callus and shoot of *R. lutea* was altered with different concentrations of ZnO-NPs. Nevertheless, the precise cause of ZnO-NPs impact on phenolic and flavonoid accumulation is still unknown. Since ZnO-NPs induce oxidative stress, which triggers the plant's antioxidant defense mechanisms, leading to more synthesis of secondary metabolites. ZnO-NPs can effectively stimulate the biosynthesis of phenolic and flavonoid compounds, augment the plant's antioxidant capacity, and potentially improve its resilience to environmental stresses. Furthermore, the flavonoid content in plants differs with ZnO-NP treatment, and generally, higher ZnO-NP concentrations prevent the synthesis of secondary metabolites. The application of 60 mg/L ZnO-NPs in MS medium, TPC and TFC content in the shoot of *R. lutea* decreased. Previous research has demonstrated that a low concentration of ZnO-NPs can increase the accumulation of total flavonoid content in dragonhead.^82^ The findings on *R. lutea* callus align with a previous study on *E. macrochaetus,*^43^ a significant increase in flavonoid content following ZnO nanoparticle treatment was observed. However, different treatment doses of ZnO-NPs positively improved the TPC and TFC in the callus stage of *R. lutea*. In another study, as performed on *Stevia rebaudiana*, low concentrations of ZnO-NPs exhibited positive effects on the synthesis of total flavonoids.^83^ The improvement of TPC and TFC in *R. lutea* at various doses of ZnO-NPs is supported by the result of Wang et al.^33^ as *G. bioloba* treated with ZnO-NPs, the leaves had an accumulation of various secondary metabolites, such as kaempferol, isorhamnetin, and total flavanol. Since the callus biomass of *R. lutea* was positively improved with a high concentration of ZnO-NPs (60 mg/L); nevertheless, it also promoted the synthesis of total phenolic and flavonoid content (Table 1). In another study, it was found that the addition of ZnO nanoparticles to the culture medium enhanced the thymol and carvacrol production in *Thymus kotschyanus* callus cultures.^84^

The application of FCM to estimate the genome size of plants after they have been manipulated *in vitro* or treated with chemicals has become a routine technique. Flow cytometry is a consistent and fast technique generally used for genome size (2 C DNA content) estimation and detection of ploidy level.^85^^,^^86^ The genetic stability was confirmed in the callus and shoot of *R. lutea,* as both regeneration stages had the same genome size (1.91 pg) without ZnO-NP treatment. In our study, no evidence of a change in genome size was observed up to 30 mg/L ZnO-NPs, indicating that no induction of polyploidy or aneuploidy. This might be due to the explant (callus and shoot) physiology, uptake pattern, ZnO-NP concentration, and duration of exposure. Moreover, the growth pattern of callus and shoot of *R. lutea* after 30 d of treatment showed similarity to the control. A non-significant variation was observed in callus and shoot of *R. lutea* with 60 mg/L ZnO-NPs (1.92 pg ± 0.02) and (1.93 pg ± 0.05) than the control (1.91 pg ± 0.00 and 1.91 pg ± 0.03), respectively, which does not indicate true instability. The enhanced biochemical attributes in callus and shoot at 60 mg/L of ZnONPs might have helped in genome stability by scavenging the generated ROS. This finding is consistent with those of previous studies^43^^,^^87^^,^^88^ who found genome size variation with various concentrations of ZnO-NPs in *Echinops macrochaetus*, *Allium cepa,* and *L*a*mprocapnos spectabilis*.

Other molecular markers, such as start codon targeted (SCoT) and sequence-related amplified polymorphism (SRAP), showed a low degree of polymorphism and confirmed the genetic stability in *Eriobotrya japonica* under ZnO-NP treatment.^62^ However, the effect of different concentrations of ZnO-NPs varies among plant species, as a study performed on *Salvadora persica* with ZnO-NPs observed genotoxicity during callus cultures.^89^ The start codon targeted polymorphism (SCoT) and randomly amplified polymorphic DNA (RAPD) markers applied on *Chrysanthemum* treated with ZnO-NPs confirmed their genetic fidelity.^90^ Thus, the present protocol under the application of zinc nanoparticles for mass propagation of *R. lutea* is safe to use since regenerants remain clonal and true-to-type with numerous potential traits. More research is necessary to comprehend how varying concentrations of biogenic ZnO-NPs impact the tissue culture of various types of explants, as plant cells at different developmental stages may respond in diverse ways.

## Conclusion

Biogenic nanoparticles have emerged as a useful tool in the development of plant tissue culture techniques, offering improved abilities to increase the growth of the plants and stimulate the production of secondary metabolites. In the present study, the application of an optimum concentration of ZnO-NPs with reduced concentrations of 2,4-D and BAP acted as a nanosupplement for the regeneration stages of *R. lutea*. It has a promoting effect on *in vitro*-raised callus and shoots of *R*. *lutea*, enhancing growth, protein content, sugar, and the production of secondary metabolites. The production of biomass was improved in both the callus and shoot compared to the control with a low concentration of ZnO-NPs. These results highlight the importance of selecting suitable concentrations of ZnO-NPs based on biochemical as well as growth attributes for plantlet regeneration. The optimized concentration of ZnO-NPs could be integrated into micropropagation protocols to improve yield and possibly prime plantlets against abiotic stresses, although further testing (such as *ex vitro* acclimatization success and field performance) is needed. Moreover, genetic stability was assessed in the regeneration stages of *R. lutea*, under low concentrations of ZnO-NPs using FCM, confirming the protocol's reliability for future studies. Further research should investigate the enhancement of flavonol glycosides, specifically kaempferol, through the application of ZnONPs, as both the total phenolic content (TPC) and the total flavonoid content (TFC) have shown improvement. Thus, synthesized bio-fabricated zinc oxide nanoparticles using *C. arvensis* leaf extract are environmentally safe and could be used for the multiplication of medicinal plant species and improvement of their yield and quality traits in tissue culture. The different concentrations of ZnO-NPs showed positive effect on growth, biochemical and phytochemical parameters as carried out on regeneration stages of *Reseda lutea* (Figure 11).

## Data Availability

All the data supporting the findings of the present study are included in this article.
